# Epitope-Specific Tolerance Modes Differentially Specify Susceptibility to Proteolipid Protein-Induced Experimental Autoimmune Encephalomyelitis

**DOI:** 10.3389/fimmu.2017.01511

**Published:** 2017-11-09

**Authors:** Lei Wang, Julia Winnewisser, Christine Federle, Gregor Jessberger, Klaus-Armin Nave, Hauke B. Werner, Bruno Kyewski, Ludger Klein, Maria Hinterberger

**Affiliations:** ^1^Institute for Immunology, Biomedical Center (BMC) Munich, Ludwig-Maximilians-University, Munich, Germany; ^2^Department of Neurogenetics, Max Planck Institute of Experimental Medicine, Goettingen, Germany; ^3^Division of Developmental Immunology, German Cancer Research Center (DKFZ), Heidelberg, Germany

**Keywords:** autoimmunity, experimental autoimmune encephalomyelitis, central tolerance, antigen presentation, thymic epithelium, epitope, ignorance, myelin proteolipid protein

## Abstract

Immunization with myelin components can elicit experimental autoimmune encephalomyelitis (EAE). EAE susceptibility varies between mouse strains, depending on the antigen employed. BL/6 mice are largely resistant to EAE induction with proteolipid protein (PLP), probably a reflection of antigen-specific tolerance. However, the extent and mechanism(s) of tolerance to PLP remain unclear. Here, we identified three PLP epitopes in PLP-deficient BL/6 mice. PLP-sufficient mice did not respond against two of these, whereas tolerance was “leaky” for an epitope with weak predicted MHCII binding, and only this epitope was encephalitogenic. In TCR transgenic mice, the “EAE-susceptibility-associated” epitope was “ignored” by specific CD4 T cells, whereas the “resistance-associated” epitope induced clonal deletion and T_reg_ induction in the thymus. Central tolerance was autoimmune regulator dependent and required expression and presentation of PLP by thymic epithelial cells (TECs). TEC-specific ablation of PLP revealed that peripheral tolerance, mediated by dendritic cells through recessive tolerance mechanisms (deletion and anergy), could largely compensate for a lack of central tolerance. However, adoptive EAE was exacerbated in mice lacking PLP in TECs, pointing toward a non-redundant role of the thymus in dominant tolerance to PLP. Our findings reveal multiple layers of tolerance to a central nervous system autoantigen that vary among epitopes and thereby specify disease susceptibility. Understanding how different modalities of tolerance apply to distinct T cell epitopes of a target in autoimmunity has implications for antigen-specific strategies to therapeutically interfere with unwanted immune reactions against self.

## Introduction

Experimental autoimmune encephalomyelitis (EAE) is an autoimmune demyelinating disease of the central nervous system (CNS) that develops in rodents after immunization with whole brain extract or purified myelin components (1, 2). It is characterized by infiltration of autoreactive CD4^+^ T cells and other inflammatory cells into the CNS. The pathophysiology and clinical symptoms of EAE, including various degrees of paralysis, to some extent resemble the human disease multiple sclerosis (MS) (3).

Depending on the CNS autoantigen employed for immunization, there is a considerable degree of variation between inbred mouse strains with regards to EAE susceptibility and disease course. For instance, B10.PL and PL/J mice are highly susceptible to disease induction with purified myelin basic protein (MBP) or particular MBP peptides, whereas many other mouse strains are resistant to MBP-induced EAE (4–6). The most commonly used encephalitogenic protocol for C57BL/6 (BL/6) mice relies on a particular myelin oligodendrocyte glycoprotein (MOG) peptide and results in a monophasic disease. Upon immunization with myelin proteolipid protein (PLP), SJL/J mice develop a fulminant form of relapsing-remitting EAE, whereas most other strains, including BL/6, are considered largely “resistant” to PLP-induced EAE (6–8).

Numerous genetic and non-genetic factors have been reported to modulate EAE susceptibility and disease severity in a complex fashion, but in many cases, the underlying molecular mechanisms remain elusive (9, 10). Among the various inheritable traits that control the susceptibility to EAE in mice and MS in humans, the MHC is the by far most prominently associated genetic locus (9–11). Moreover, it is well established that the magnitude of the *in vitro* CD4 T cell response to myelin antigens in classical immunization recall experiments is a robust correlate of disease susceptibility. For instance, PLP-EAE susceptible SJL mice display a vigorous CD4 T cell response upon immunization with PLP protein or particular pools of PLP-peptides, whereas resistant strains such as BL/6, BALB/c, or CBA exhibit a much weaker response (7, 8). Although none of the strains that are susceptible to EAE induction with a given CNS protein develop spontaneous disease, it is undisputed that the composition and responsiveness of their CD4 T cell compartment is a critical determinant of disease susceptibility.

CD4 T cells reactive to MBP or PLP are constituents of the normal human T cell repertoire (12–14). Limitations inherent to human studies so far preclude a conclusive assessment whether this in fact indicates the absence of antigen-specific tolerance or whether these autoreactive cells represent a residual fraction of the repertoire that has escaped tolerance induction. However, a precise understanding of how different modalities of tolerance shape the T cell reactivity to CNS autoantigens and how recessive modes of tolerance, i.e., deletion and anergy, or dominant, i.e., Treg-mediated, tolerance cooperate and/or differentially apply to distinct T cell epitopes of a target in autoimmunity has implications for strategies that aim to therapeutically interfere with unwanted immune reactions against the CNS.

Mice lacking particular CNS autoantigens have been used to assess whether the magnitude and quality of the response to a given myelin protein is influenced by antigen-specific tolerance. MOG-specific CD4 T cell responses were found to be identical between *Mog*^+/+^ and *Mog*^–/–^ mice, and it was suggested that this apparent lack of tolerance to MOG explains the high pathogenicity of the anti-MOG immune response in several mouse strains (15). By contrast, *Mbp*^+/+^ mice on the C3H or BALB/c background displayed a substantially weaker CD4 T cell response to MBP immunization than MBP-deficient mice, indicating that their T cell repertoire is somehow curtailed by endogenously expressed MBP (16, 17). Using PLP knockout mice, we demonstrated that the relatively moderate response of PLP-sufficient BL/6 mice to immunization with PLP is a consequence of specific tolerance rather than the reflection of an inherently low responsiveness (8).

The evidence for specific tolerance to the myelin antigens MBP and PLP is at odds with the originally held view that CNS proteins are topologically sequestered from the immune system. However, the idea that myelin antigens are exclusively expressed behind the blood–brain barrier, and thereby excluded from tolerance induction, has been largely abandoned (1). In case of PLP, mRNA transcripts are readily detectable in primary and secondary lymphoid organs (18, 19), and thymus chimeras revealed that the physiological expression of PLP in thymic epithelial cells (TECs) was sufficient to recapitulate the diminished CD4 response of PLP-sufficient BL/6 mice (8).

CD4 T cell tolerance to PLP in the “PLP-EAE-resistant” BL/6 strain has so far only been “operationally” defined as a reduced polyclonal CD4 T cell recall response upon immunization compared to PLP-deficient mice. However, it was unclear through which cellular mechanism(s), that is, clonal deletion and/or diversion into the Foxp3^+^ T_reg_ lineage, tolerance is achieved, and whether these modes of tolerance similarly apply to distinct epitopes of PLP. Furthermore, it remained open whether intrathymic PLP expression was “essential” for tolerance to PLP, both in terms of its impact on the fate of PLP-specific CD4 T cells as well as disease susceptibility. In the present study, we employed two novel strains of TCR transgenic mice in conjunction with strategies to selectively interfere with PLP expression or presentation in distinct cellular compartments to obtain a comprehensive view of the modalities of CD4 T cell tolerance to PLP.

## Results

### PLP Epitopes and Epitope-Specific EAE Susceptibility in BL/6 Mice

**Figure 1:**
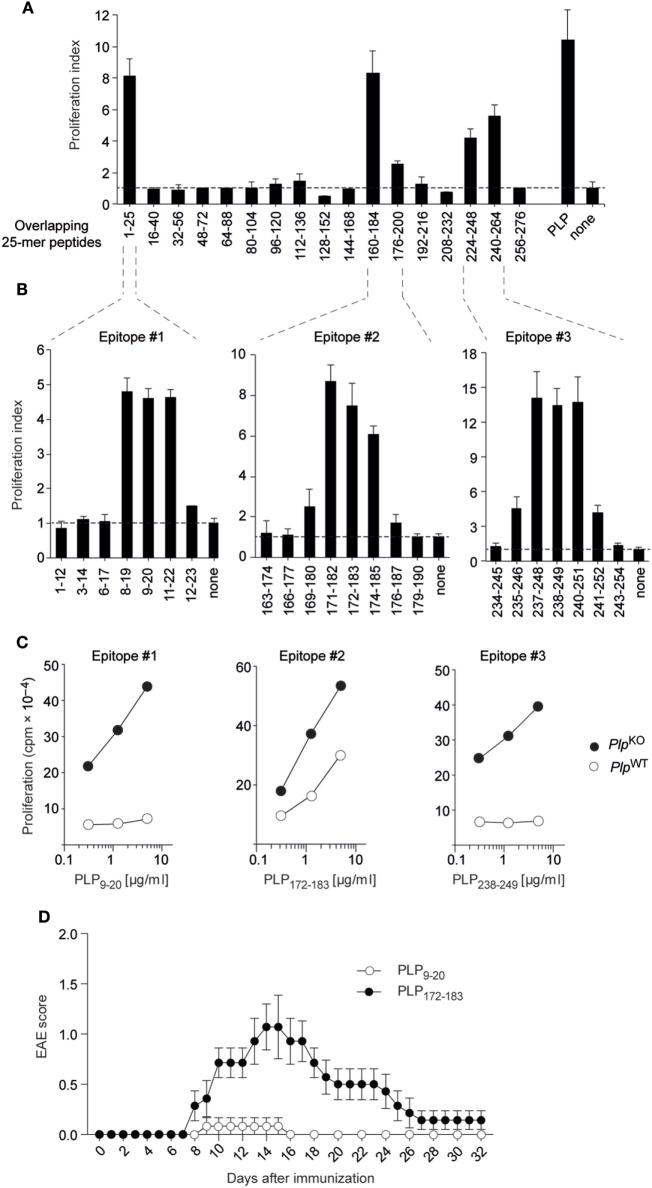
Proteolipid protein (PLP) epitopes and epitope-specific experimental autoimmune encephalomyelitis (EAE) susceptibility in BL/6 mice. **(A)** *Plp*^KO^ BL/6 mice were immunized with PLP protein. Nine days later, draining lymph node cells were re-stimulated *in vitro* with overlapping 25-mers spanning the entire PLP protein. Responses to peptides are shown as proliferation indices. **(B)** Fine mapping of epitopes with overlapping 12-mer peptides. **(C)** CD4 T cell recall response of *Plp*^KO^ or *Plp*^WT^ mice upon immunization with 12-mer peptides containing the core epitopes #1, #2, and #3. **(D)** EAE symptoms in *Plp*^WT^ mice upon immunization with the 12-mer peptides PLP_9–20_ (*n* = 6) or PLP_172–183_ (*n* = 7) together with administration of pertussis toxin on days 0 and 2. Data in panels **(A–C)** are from individual mice representative for *n* ≥ 5 each.

We initially broadly defined the targets of CD4 T cell reactivity to PLP in “non-tolerant” *Plp*^KO^ BL/6 mice through re-stimulating lymph node T cells from immunized mice with a set of overlapping 25-mer peptides corresponding to the entire PLP protein (Figure 1A) (8). The PLPko mouse used in our study has been characterized extensively with regards to a verification of the absence of PLP protein or any truncated version thereof (20). Overlapping 12-mer peptides spanning these regions were then used to precisely fine-map the respective I-A^b^-restricted epitopes (Figure 1B). This revealed three core 9-mer epitopes spanning amino acids 11–19, 174–182, and 240–248, in the following referred to as epitopes #1, #2, and #3, respectively. The experimental epitope definition was retrospectively combined with an *in silico* prediction of T cell epitopes using the *Immune Epitope Database and Analysis Resource* (IEDB) (21, 22). The IEDB algorithm predicts and ranks the relative binding strengths of all 15-mer peptides that can be generated from a given protein. For PLP, the seven 15-mer peptides containing epitope #3 were among the top eight predicted I-A^b^ binders, and all of the 15-mers harboring epitope #1 were ranked between positions 10 and 20 (Figure S1 in Supplementary Material). Epitope #2-containing 15-mers had the weakest binding scores and ranked between positions 33 and 57. Consistent with this relative ranking, an *in silico* prediction of MHC-binding affinities using the SSM-align algorithm (23) yielded mean IC_50_ values of 168 ± 61 nM for epitope #3-containing peptides and 715 ± 262 or 1,533 ± 498 nM for peptides containing epitopes #1 or #2, respectively.

Compared to the pronounced CD4 T cell responses to all three epitopes in *Plp*^KO^ mice, there was essentially no recall response to epitopes #1 and #3 with cells from immunized *Plp*^WT^ mice (Figure 1C). However, a robust response to epitope #2 was observed, although the magnitude of this response was somewhat attenuated as compared to *Plp*^KO^ mice. To assess how the absence or presence of CD4 T cell responsiveness to these PLP epitopes correlated with EAE susceptibility, we immunized *Plp*^WT^ mice with epitopes #1, #2, or #3 according to the EAE protocol. Whereas “EAE-immunization” with epitope #1 and epitope #3 failed to promote disease symptoms, epitope #2 elicited EAE with a typical acute disease course, that is, onset of symptoms at around day 8, followed by a disease peak at days 14–16 and subsequent remission (Figure 1D, and data not shown).

Together, these observations reveal a differential impact of self-tolerance on CD4 T cell responses to three distinct epitopes of PLP in BL/6 mice. Tolerance was “leaky” for the epitope with the lowest predicted MHC II-binding capacity. Moreover, the absence or presence of recall responsiveness to a given epitope tightly correlated with resistance or susceptibility to EAE, respectively.

### “Ignorance” toward PLP Epitope #2

**Figure 2:**
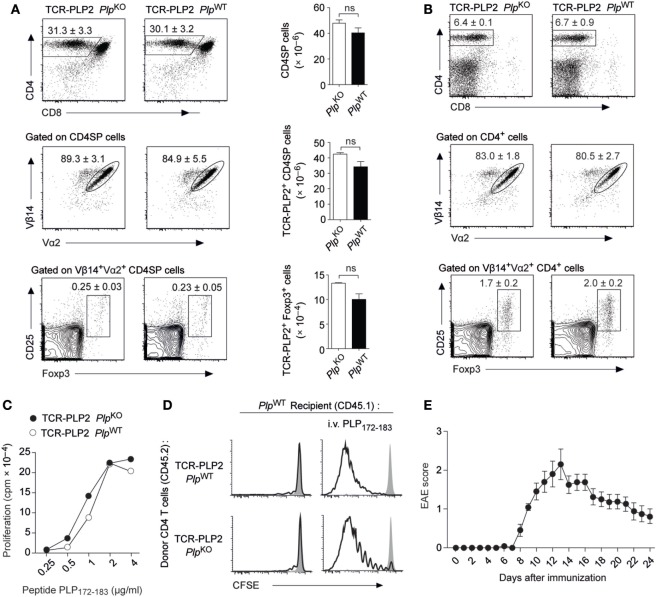
“Ignorance” toward proteolipid protein (PLP) epitope #2 in TCR-PLP2 transgenic mice. **(A)** Thymocyte subset composition of TCR-PLP2 transgenic mice on *Plp*^KO^ or *Plp*^WT^ background. The average frequency ± SEM of CD4SP cells (upper plot), TCR-PLP2^+^ cells (Vα2^+^Vβ14^+^) among gated CD4SP thymocytes (middle), and CD25^+^Foxp3^+^ cells among gated TCR-PLP2^+^ CD4SP cells is indicated (*n* ≥ 8 per genotype). The bar diagrams on the right show absolute cell numbers of the respective subsets. **(B)** Peripheral phenotype of TCR-PLP2 transgenic mice on *Plp*^KO^ or *Plp*^WT^ background. The average frequency ± SEM of CD4 T cells (upper plot), TCR-PLP2^+^ cells (Vα2^+^Vβ14^+^) among gated CD4 T cells (middle) and CD25^+^Foxp3^+^ cells among gated TCR-PLP2^+^ CD4 T cells (lower) are indicated (*n* ≥ 8 per genotype). **(C)** Proliferation of purified CD4 T cells from TCR-PLP2 transgenic mice on *Plp*^KO^ or *Plp*^WT^ background upon *in vitro* stimulation with PLP_172–183_. Data are from individual mice representative for *n* ≥ 3 each. **(D)** CFSE-labeled CD4 T cells from *Plp*^KO^ or *Plp*^WT^ TCR-PLP2 transgenic mice (CD45.2) were i.v. injected into *Plp*^WT^ recipients (CD45.1) that had (right) or had not (left) been immunized with PLP_172–183_ the day before. Four days later, lymph node cells were harvested and analyzed by FACS. Histograms show the CFSE intensity of gated CD4^+^CD45.2^+^ cells. The filled histogram overlay is from undivided control cells in a *Plp*^KO^ recipient. Data are representative of three experiments with *n* = 5 each. **(E)** Experimental autoimmune encephalomyelitis (EAE) disease course in TCR-PLP2 *Plp*^WT^ mice upon immunization with the 12-mer peptide PLP_172–183_ together with administration of pertussis toxin on days 0 and 2 (*n* = 14).

In order to follow the fate of epitope #2-specific CD4 T cells and characterize the apparent “failure” of tolerance induction to this epitope, we generated transgenic mice (TCR-PLP2) expressing a PLP_172–183_-specific TCR that was derived from immunized *Plp*^KO^ mice. In TCR-PLP2 *Plp*^KO^ mice, i.e., in the absence of cognate self-antigen, there was a pronounced skewing toward the CD4 lineage in the thymus, and the vast majority of CD4 single-positive (SP) thymocytes expressed the transgenic TCR chains (Vα2 and Vβ14) (Figure 2A). A very small fraction of Vα2^+^Vβ14^+^ CD4 SP cells expressed Foxp3. Importantly, none of these parameters was altered in TCR-PLP2 *Plp*^WT^ mice, indicating that PLP epitope #2-specific CD4 T cells were not subject to central tolerance induction (Figure 2A). Also in the periphery, neither the frequency of CD4 T cells nor their expression of Vα2^+^Vβ14^+^ was different between TCR-PLP2 mice on *Plp*-deficient or -sufficient background, suggesting that epitope #2-specific T cells in both settings persisted as naïve cells (Figure 2B). Consistent with this, purified CD4 T cells from TCR-PLP2 *Plp*^WT^ displayed an identical *in vitro* proliferative response to stimulation with titrated amounts of PLP_172–183_ as cells from TCR-PLP2 *Plp*^KO^ controls (Figure 2C). Adoptively transferred naïve TCR-PLP2 cells from *Plp*^KO^ mice persisted in a non-dividing state in *Plp*^WT^ recipients, whereas cells from both TCR-PLP2 *Plp*^KO^ or TCR-PLP2 *Plp*^WT^ origin proliferated upon challenge with cognate peptide, irrespective of being parked in a *Plp*-deficient or -sufficient recipient (Figure 2D). These findings further substantiated the idea that PLP epitope #2 of natural origin is not sufficiently processed and presented from physiological sources to become “visible” to peripheral CD4 T cells. However, “EAE-immunization” with peptide PLP_172–183_ elicited EAE in TCR-PLP2 *Plp*^WT^ mice, suggesting that these TCR transgenic cells can turn into pathogenic effectors upon appropriate stimulation and recognize the physiologically processed epitope (Figure 2E).

Thus, PLP_172–183_-specific TCR transgenic CD4 T cells with encephalitogenic potential were not subject to tolerance induction. Instead, they persisted in a state of “ignorance” that was broken upon immunogenic delivery of cognate antigen, in line with the capacity of PLP_172–183_ immunization to elicit EAE in a polyclonal setting.

### Spontaneous EAE in TCR-PLP2 Rag1^–/–^ Mice

**Figure 3:**
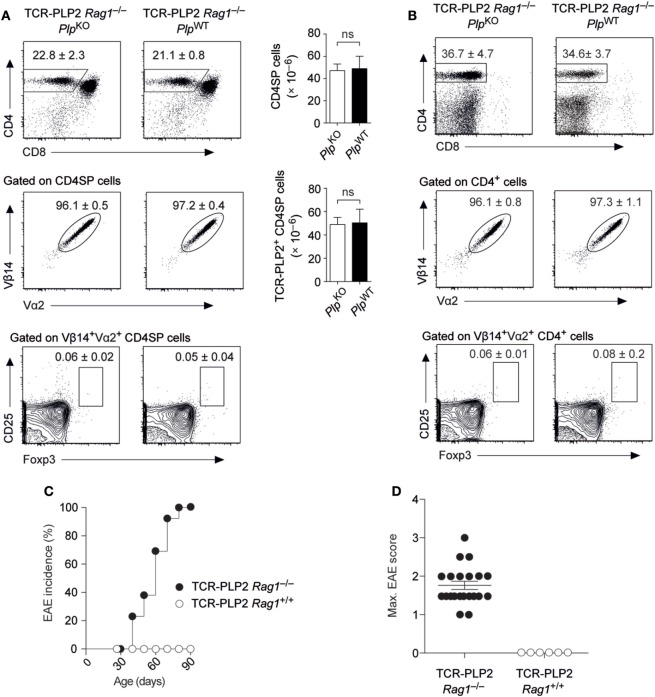
Spontaneous experimental autoimmune encephalomyelitis (EAE) in TCR-PLP2 transgenic mice on *Rag1*-deficient background. **(A)** Thymocyte subset composition of TCR-PLP2 transgenic *Rag1*^–/–^ mice on *Plp*^KO^ or *Plp*^WT^ background. The average frequency ± SEM of CD4SP cells (upper plot), TCR-PLP2^+^ cells (Vα2^+^Vβ14^+^) among gated CD4SP thymocytes (middle) and CD25^+^Foxp3^+^ cells among gated TCR-PLP2^+^ CD4SP cells (lower) is indicated (*n* ≥ 9 each). The bar diagrams on the right show absolute cell numbers of the respective subsets. **(B)** Peripheral phenotype of TCR-PLP2 transgenic mice on *Plp*^KO^ or *Plp*^WT^ background. The average frequency ± SEM of CD4 T cells (upper plot), TCR-PLP2^+^ cells (Vα2^+^Vβ14^+^) among gated CD4 T cells (middle) and CD25^+^Foxp3^+^ cells among gated TCR-PLP2^+^ CD4 T cells (lower) are indicated (*n* ≥ 9 each per genotype). **(C,D)** Cumulative incidence and maximum disease score of spontaneous EAE in TCR-PLP2 *Rag1*^–/–^ (*n* = 21) and TCR-PLP2 *Rag1*^+/+^ mice (*n* = 6).

The expression of an endogenously rearranged TCRα chain together with the transgenic TCR elements may result in a dual specificity and thereby complicate the interpretation of TCR instructed cell fate decisions. For instance, the emergence of a small population of Foxp3^+^ cells among Vα2^+^Vβ14^+^ cells in both thymus and periphery of TCR-PLP2 mice was PLP independent, and thus likely to reflect a “cryptic autoreactivity” conveyed by endogenous TCR rearrangements. To assess the consequences of excluding endogenous TCR rearrangements, we generated *Rag1*-deficient TCR-PLP2 mice. Irrespective of an intact *Plp* gene, this resulted in the virtual absence of Foxp3^+^ cells from thymus and periphery. Importantly, as in *Rag1*^+/+^ mice, the frequency and absolute number TCR-PLP2^+^ cells was indistinguishable between *Plp*-deficient or –sufficient mice, confirming that their cognate antigen PLP_172–183_ is essentially “invisible” to these cells (Figures 3A,B). However, in contrast to mice on *Rag1*^+/+^ background, PLP-sufficient TCR-PLP2 Rag1^–/–^ mice developed “spontaneous” EAE starting at around 5 weeks of age (Figures 3C,D).

These observations confirmed that TCR-PLP2^+^ CD4 T cells can be pathogenic, yet do not encounter cognate antigen during thymic differentiation or in the periphery, so that they initially remain in a state of “ignorance.” However, in a truly monoclonal setting their latent autoimmune potential can with increasing age be “spontaneously” unleashed, possibly facilitated by the lack of T_reg_ cells.

### Central Tolerance toward PLP Epitope #1

**Figure 4:**
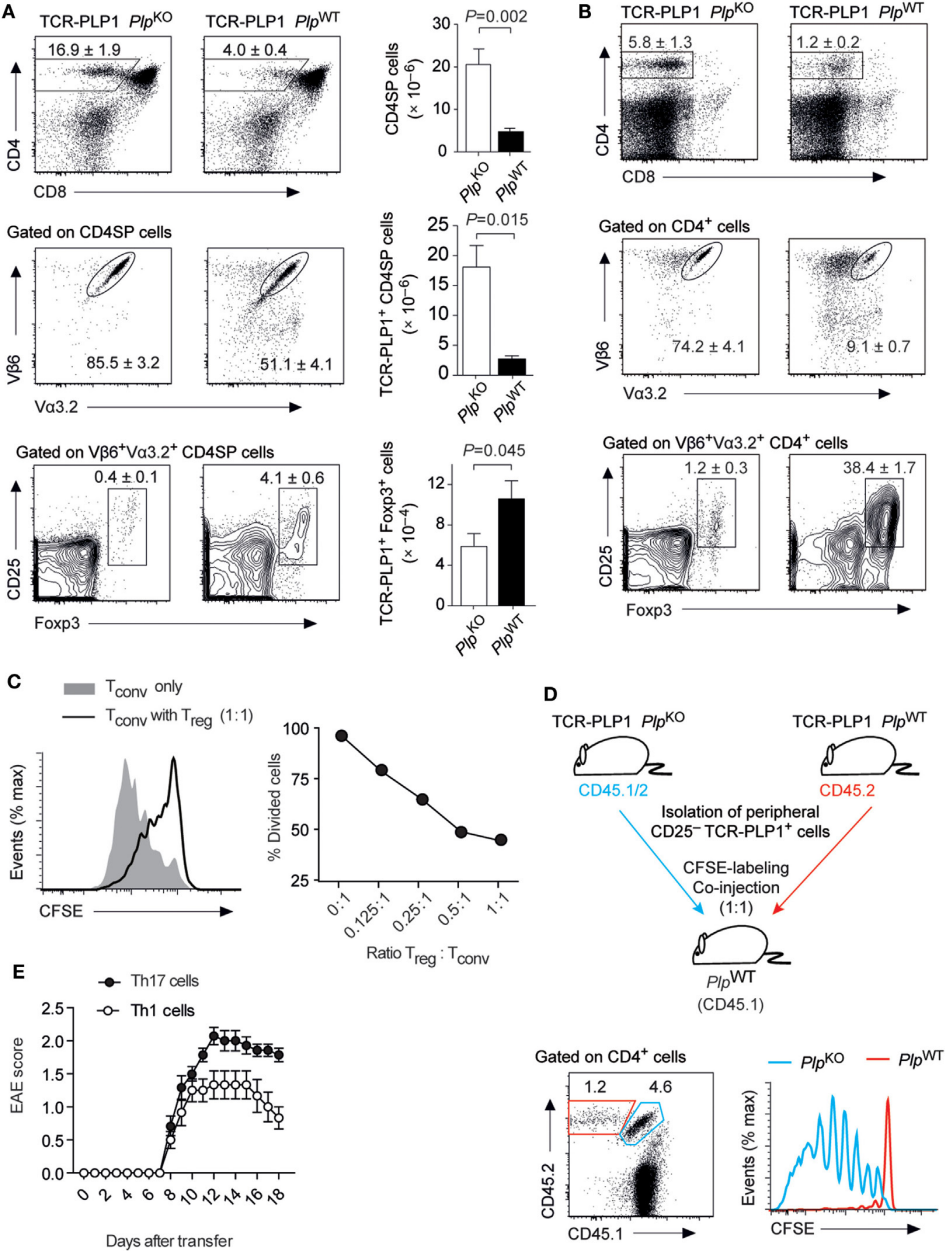
Central tolerance to proteolipid protein (PLP) epitope #1 in TCR-PLP1 transgenic mice. **(A)** Thymocyte subset composition of TCR-PLP1 transgenic mice on *Plp*^KO^ or *Plp*^WT^ background. The average frequency ± SEM of CD4SP cells (upper plot), TCR-PLP1^+^ cells (Vα3.2^+^Vβ6^+^) among gated CD4SP thymocytes (middle) and CD25^+^Foxp3^+^ cells among gated TCR-PLP1^+^ CD4SP cells is indicated (*n* ≥ 8 mice per genotype). The bar diagrams on the right show absolute cell numbers of the respective subsets. **(B)** Peripheral phenotype of TCR-PLP1 transgenic mice on *Plp*^KO^ or *Plp*^WT^ background. The average frequency ± SEM of CD4 T cells (upper plot), TCR-PLP1^+^ cells (Vα3.2^+^Vβ6^+^) among gated CD4 T cells (middle) and CD25^+^Foxp3^+^ cells among gated TCR-PLP1^+^ CD4 T cells (lower) are indicated (*n* ≥ 8 mice per genotype). **(C)** Purified naïve TCR-PLP1^+^ CD4 T cells from TCR-PLP1 *Plp*^KO^ mice were labeled with CFSE and cultured *in vitro* with irradiated splenoctyes and peptide PLP_9–20_ in the presence or absence of titrated numbers of TCR-PLP1^+^CD25^+^ CD4 T cells from TCR-PLP1 *Plp*^WT^ mice. The histogram overlay shows a representative CFSE profile on day 3 of cells when cultured alone (gray histogram) or with an equal number of T_reg_ cells (open histogram). The diagram on the right shows the dose/response curve with different ratios of T_reg_ cells. **(D)** Purified TCR-PLP1^+^CD25^–^ CD4 T cells from TCR-PLP1 mice on *Plp*^WT^ (CD45.2) or *Plp*^KO^ background (CD45.1/2) were labeled with CFSE and co-injected i.v. into *Plp*^WT^ (CD45.1) recipients. Three days after transfer, lymph node cells were harvested and stained for CD45.1 and CD45.2. The dot plot on the left shows the relative abundance of cells of *Plp*^WT^ (CD45.2; red gate) or *Plp*^KO^ origin (CD45.1/2; blue gate). The histogram overlay on the right shows the CFSE profile of the respective cells. Data are representative of two independent experiments with *n* ≥ 3 each. **(E)** Disease course of “adoptive” experimental autoimmune encephalomyelitis (EAE) in *Rag1*^–/–^ recipients after transfer of Th1- or Th17-polarized TCR-PLP1^+^ CD4 T cells from TCR-PLP1 *Plp*^KO^ mice.

In contrast to our observations with the “EAE-susceptibility-associated” epitope #2, BL/6 mice did not display a CD4 T cell recall response to epitope #1 and were resistant to EAE induction by immunization with this peptide (see Figure 1). In order to characterize the mode of CD4 T cell tolerance induction to the “EAE-resistance-associated” epitope #1, we generated a PLP_11–19_-specific TCR transgenic mouse strain (TCR-PLP1). Compared to TCR-PLP1 *Plp*^KO^ controls, the frequency and number of CD4 SP cells in the thymus of TCR-PLP1 *Plp*^WT^ mice were strongly diminished (Figure 4A). Moreover, whereas in PLP-deficient mice, around 85% of CD4 SP cells expressed both transgenic TCR chains, only about 50% of CD4 SP cells were Vα3.2^+^Vβ6^+^ in PLP-sufficient mice. Among the remaining TCR-PLP1^+^ CD4 SP cells in PLP^WT^ mice, an elevated frequency of cells was Foxp3^+^, whereby in terms of absolute numbers, this reflected an only moderate, yet significant, increase (Figure 4A). In peripheral lymphoid organs of TCR-PLP1 *Plp*^WT^ mice, the proportion of CD4^+^ T cells was reduced by about fourfold when compared to *Plp*^KO^ controls (Figure 4B). Whereas in PLP-deficient mice, around 75% of peripheral CD4 T cells expressed the transgenic TCR, less than 10% of peripheral CD4^+^ T cells did so in TCR-PLP1 *Plp*^WT^ mice. TCR-PLP1^+^ CD4 T cells contained only a minute fraction of Foxp3^+^ cells in PLP-deficient mice, while Foxp3^+^ cells constituted more than a third of TCR-PLP1^+^ cells in *Plp*^WT^ mice (Figure 4B).

To ask whether the segregation of peripheral TCR-PLP1^+^ cells in PLP-sufficient mice into Foxp3^+^ and Foxp3^–^ cells reflected the co-existence of T_reg_ cells and bona fide naïve cells, we assessed the functional properties of both subsets. Purified CD25^+^ cells indeed suppressed the proliferation of naïve TCR-PLP1 T_conv_ cells in an antigen-specific manner (Figure 4C). However, whereas naïve TCR-PLP1 cells from PLP-deficient donors vigorously proliferated upon adoptive transfer into *Plp*^WT^ recipients, illustrating that I-A^b^/PLP_11–19_ ligands of physiological origin are constitutively “visible” in peripheral lymphoid tissues, co-transferred CD25^–^ TCR-PLP1^+^ cells from TCR-PLP1 *Plp*^WT^ mice did not proliferate, indicating that these cells were in a cell intrinsic refractory state of anergy (Figure 4D).

We did not observe spontaneous EAE in TCR-PLP1 *Plp*^WT^ mice. In order to assess the “autoimmune potential” of TCR-PLP1 CD4 T cells, we generated TCR-PLP1 *Rag*1^–/–^ *Plp*^KO^ mice. Monoclonal TCR-PLP1 CD4 T cells from these donors were differentiated *in vitro* into Th1 or Th17 effectors and subsequently transferred into *Rag*1^–/–^ *Plp*^WT^ recipients, revealing that both Th1- or Th17-polarized cells induced EAE, albeit with a slightly lower maximal disease score in the case of Th1 cells (Figure 4E).

In sum, these data showed that PLP epitope #1-specific CD4 T cells bear autoimmune potential, which was at least in part harnessed through tolerance induction during intrathymic T cell differentiation. The predominant mode of central tolerance induction among TCR-PLP1 CD4 T cells appeared to be clonal deletion. At the same time, a fraction of cells differentiated into T_reg_ cells and efficiently seeded peripheral lymphoid organs, were they co-existed with anergic Foxp3^–^CD25^–^ TCR-PLP1 cells.

### TEC-Derived PLP and Autoimmune Regulator (Aire) Are Crucial for Central Tolerance to Epitope #1

**Figure 5:**
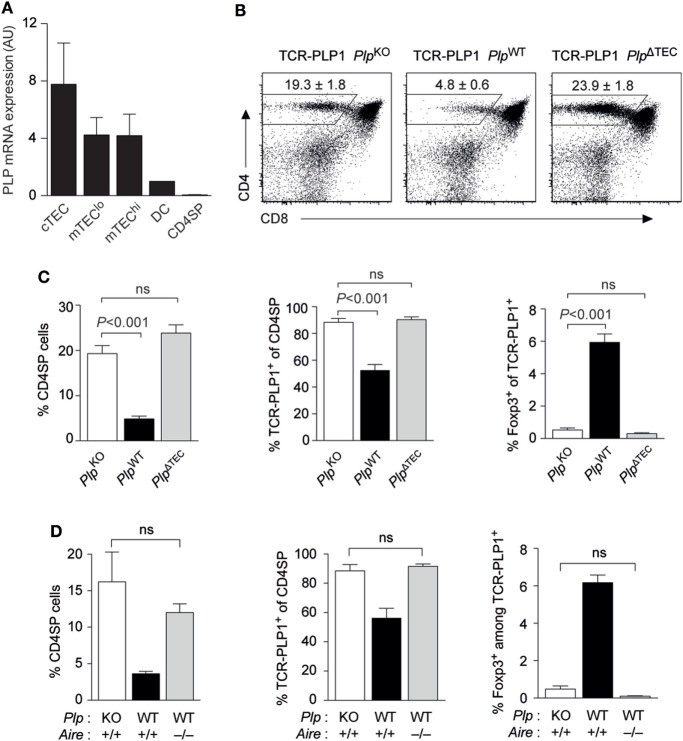
Thymic epithelial cell (TEC)-derived proteolipid protein (PLP) and autoimmune regulator (Aire) are crucial for central tolerance to epitope #1. **(A)** Relative abundance of PLP mRNA in different subsets of TECs, thymic dendritic cells (DCs), and CD4 single-positive (SP) cells. Values are indicated as arbitrary units (AU) normalized to expression in DCs. Data are representative of two independent experiments with pooled cells from ≥3 mice. **(B)** CD4 versus CD8 staining of thymus cells from TCR-PLP1 transgenic mice on *Plp*^KO^, *Plp*^WT^, or *Plp*^ΔTEC^ background. **(C)** Average frequency ± SEM of CD4SP cells (left), TCR-PLP1^+^ cells (Vα3.2^+^Vβ6^+^) among gated CD4SP thymocytes (middle) and CD25^+^Foxp3^+^ cells among gated TCR-PLP1^+^ CD4SP cells (right) in TCR-PLP1 mice on *Plp*^KO^, *Plp*^WT^, or *Plp*^ΔTEC^ background (*n* = 4 each). **(D)** Average frequency ± SEM of CD4SP cells (left), TCR-PLP1^+^ cells (Vα3.2^+^Vβ6^+^) among gated CD4SP thymocytes (middle), and CD25^+^Foxp3^+^ cells among gated TCR-PLP1^+^ CD4SP cells (right) in TCR-PLP1 mice on *Plp*^KO^ (*n* = 3), *Plp*^WT^ (*n* = 3), or *Plp*^WT^ *Aire*^–/–^ (*n* = 6) background.

We previously reported that *Plp*^WT^ → *Plp*^KO^ thymus chimeras, where TECs are the sole potential source of tolerizing antigen, recapitulated the “operational tolerance” of *Plp*^WT^ mice, as evident from a similar reduction of the CD4 T cell recall response after immunization compared to *Plp*^KO^ mice (8). This indicated that PLP expression in TECs was sufficient for CD4 T cell tolerance. Given that PLP transcripts are also detectable in dendritic cells (DCs), albeit at substantially lower levels (Figure 5A), we wondered whether TEC-derived PLP was in fact essential for central tolerance, or whether there was a degree of redundancy with other potential intra- or extrathymic sources of tolerizing antigen. To address this, we generated mice with a conditional deletion of the *Plp* gene in TECs (Foxn1-Cre *Plp*^floxed^), in the following referred to as *Plp*^ΔTEC^. Strikingly, all manifestations of central tolerance in the TCR-PLP1 system were abolished in *Plp*^ΔTEC^ mice (Figures 5B,C). Thus, both the frequency of CD4 SP cells as well as the proportion of Vα3.2^+^Vβ6^+^ among CD4 SP cells were indistinguishable from those observed in the complete absence of PLP in *Plp*^KO^ mice. In the same vein, the frequency of Foxp3^+^ cells among TCR-PLP1 CD4 SP cells of *Plp*^ΔTEC^ mice was identical to that in “antigen-free” *Plp*^KO^ mice.

The “promiscuous” transcription of peripheral antigens in the thymus is mostly confined to “mature” medullary thymic epithelial cells (mTECs) expressing high levels of MHCII, and this reflects the predominant expression of the transcriptional activator Aire in this cell type. PLP’s intrathymic expression pattern is rather untypical for a promiscuously expressed tissue-restricted antigen in that it is also detectable in “immature” (MHCII^lo^) mTECs and at comparable levels also in cortical thymic epithelial cells (cTECs) (Figure 5A). Since MHCII^lo^ mTECs and cTECs barely, or not at all, express Aire, respectively, we hypothesized that central tolerance to PLP was Aire independent. However, both negative selection and T_reg_ induction were abrogated in Aire-deficient TCR-PLP1 *Plp*^WT^ mice, so that the thymocyte composition in these mice phenocopied that in TCR-PLP1 *Plp*^KO^ and TCR-PLP1 *Plp*^ΔTEC^ mice (Figure 5D).

Together, these data showed that PLP expression in TECs was necessary and sufficient for central tolerance induction in PLP epitope #1-specific CD4 T cells. Intriguingly, although PLP transcripts were not confined to Aire-expressing mTEC^hi^ cells, intrathymic tolerance induction to PLP depended upon Aire.

### Central PLP-Tolerance Is DC Independent and Requires Presentation by mTECs

Self-antigens that are expressed in TECs may be presented to developing CD4 T cells *via* two distinct, yet mutually not exclusive routes (24). On the one hand, tolerogenic encounter of such antigens by CD4 T cells may depend upon “antigen handover” and presentation by thymic DCs. On the other hand, mTECs, or TECs in general, may autonomously present endogenously expressed antigen to CD4 T cells *via* unconventional MHC class II-loading pathways (25).

**Figure 6:**
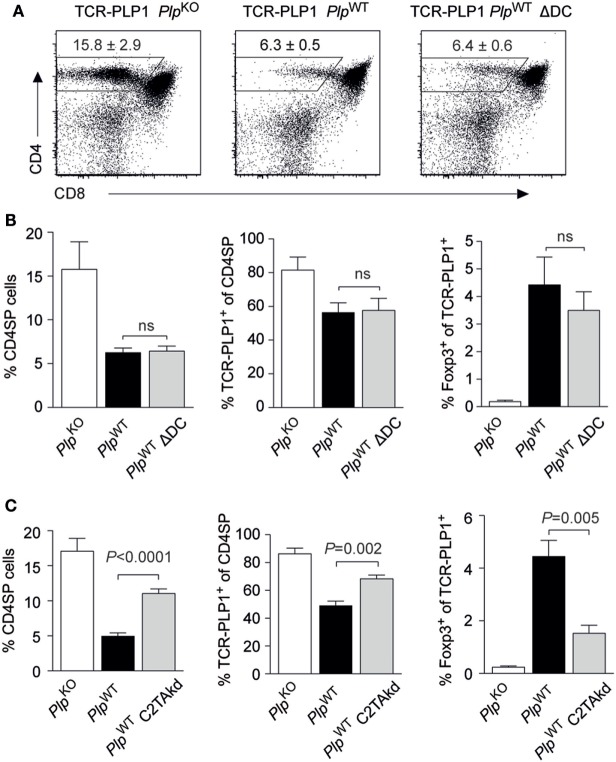
Central proteolipid protein (PLP) tolerance is dendritic cell (DC) independent and requires direct presentation by medullary thymic epithelial cells. **(A)** CD4 versus CD8 staining of thymus cells from TCR-PLP1 transgenic mice on *Plp*^KO^, *Plp*^WT^, or *Plp*^WT^ ΔDC background. **(B)** Average frequency ± SEM of CD4SP cells (left), TCR-PLP1^+^ cells (Vα3.2^+^Vβ6^+^) among gated CD4SP thymocytes (middle) and CD25^+^Foxp3^+^ cells among gated TCR-PLP1^+^ CD4SP cells (right) in TCR-PLP1 mice on *Plp*^KO^ (*n* = 3), *Plp*^WT^ (*n* = 7), or *Plp*^WT^ ΔDC (*n* = 7) background. **(C)** Average frequency ± SEM of CD4SP cells (left), TCR-PLP1^+^ cells (Vα3.2^+^Vβ6^+^) among gated CD4SP thymocytes (middle) and CD25^+^Foxp3^+^ cells among gated TCR-PLP1^+^ CD4SP cells (right) in TCR-PLP1 mice on *Plp*^KO^ (*n* = 6), *Plp*^WT^ (*n* = 8), or *Plp*^WT^ C2TAkd (*n* = 5) background.

Two experimental systems were employed to address this issue in the TCR-PLP1 model. First, we generated TCR-PLP1 *Plp*^WT^ mice lacking DCs as a result of diphtheria toxin expression in CD11c^+^ cells (“ΔDC” mice) (26). Despite the lack of DCs, the thymic phenotype of these mice was essentially identical to that of DC-sufficient TCR-PLP1 *Plp*^WT^ mice (Figures 6A,B). Second, we generated TCR-PLP1 mice with an impaired capacity to directly present antigen on mTECs as a consequence of mTEC-specific knock-down of MHC class II (“C2TAkd” mice) (27). All manifestations of central tolerance induction to PLP epitope #1 were significantly attenuated in these mice. Specifically, the proportion of CD4 SP cells, the percentage of Vα3.2^+^Vβ6^+^ among CD4 SP cells and the relative abundance of TCR-PLP1^+^ Foxp3^+^ CD4 SP cells were all partially “rescued,” i.e., they ranged in between TCR-PLP1 *Plp*^WT^ and TCR-PLP1 *Plp*^KO^ mice (Figure 6C).

Collectively, these findings documented a non-redundant role of mTECs for central tolerance induction to PLP epitope #1 through direct presentation of endogenously derived antigen. Intrathymic presentation by DCs, if occurring at all, was dispensable for central tolerance.

### Peripheral Tolerance in *Plp*^ΔTEC^ Mice

Although the conditional deletion of PLP in TECs abolished central tolerance to PLP epitope #1, neither *Plp*^ΔTEC^ mice nor TCR-PLP1 transgenic *Plp*^ΔTEC^ mice developed spontaneous EAE (data not shown). Even the active immunization of these mice with the epitope #1-containing peptide PLP_11–20_ according to the “EAE protocol” failed to promote disease symptoms (data not shown), suggesting that peripheral tolerance mechanisms were able to compensate for the absence of intrathymic censorship.

**Figure 7:**
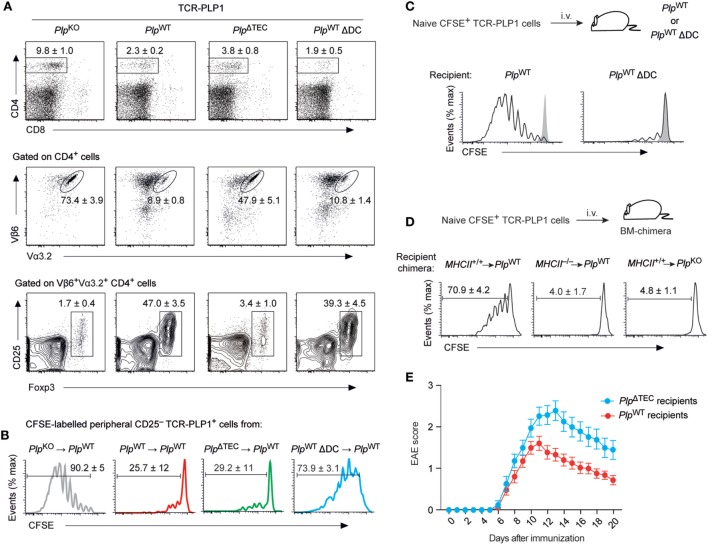
Phenotype and functionality of TCR-PLP1 cells in *Plp*^ΔTEC^ and Δdendritic cell (DC) mice. **(A)** Peripheral phenotype of TCR-PLP1 transgenic mice on *Plp*^KO^ (*n* = 8), *Plp*^WT^ (*n* = 9), *Plp*^ΔTEC^ (*n* = 3), or *Plp*^WT^ ΔDC (*n* = 7) background. The average frequency ± SEM of CD4 T cells (upper plot), TCR-PLP1^+^ cells (Vα3.2^+^Vβ6^+^) among gated CD4 T cells (middle) and CD25^+^Foxp3^+^ cells among gated TCR-PLP1^+^ CD4 T cells (lower) are indicated. **(B)** Purified TCR-PLP1^+^CD25^–^ CD4 T cells from TCR-PLP1 mice on *Plp*^KO^, *Plp*^WT^, *Plp*^ΔTEC^, or *Plp*^WT^ ΔDC background (all CD45.2) were labeled with CFSE and injected i.v. into *Plp*^WT^ (CD45.1) recipients. Three days after transfer, lymph node cells were harvested and CFSE dilution was assessed on gated CD45.2^+^ cells. Data are representative of *n* ≥ 4. **(C)** Purified TCR-PLP1^+^CD25^–^CD4 T cells from TCR-PLP1 mice on *Plp*^KO^ background (CD45.1) were labeled with CFSE and injected i.v. into *Plp*^WT^ or *Plp*^WT^ ΔDC recipients (CD45.2). Three days after transfer, lymph node cells were harvested and CFSE dilution was assessed on gated CD45.2^+^ cells. The gray histogram shows the CFSE-intensity of cells transferred into control *Plp*^KO^ recipients. Data are representative of *n* ≥ 6. **(D)** Purified TCR-PLP1^+^ CD4 T cells from TCR-PLP1 mice on *Plp*^KO^ background (CD45.1) were labeled with CFSE and i.v. injected into bone marrow (BM)-chimeric recipients (MHCII^+/+^ → *Plp*^WT^ controls, MHCII^–/–^ → *Plp*^WT^, and MHCII^+/+^ → *Plp*^KO^) (CD45.2). Three days after transfer, lymph node cells were harvested and CFSE dilution was assessed on gated CD45.2^+^ cells. **(E)** Experimental autoimmune encephalomyelitis (EAE) disease course in male *Plp*^WT^ or *Plp*^ΔTEC^ mice (non-TCR-transgenic) that had been injected i.v. with 5 × 10^6^ TCR-PLP1^+^ T cells from TCR-PLP1 *Plp*^KO^ mice 6 h before administration of PLP_9–20_ in CFA. Data are from *n* ≥ 14 each.

Indeed, the CD4 T cell compartment in secondary lymphoid tissues of TCR-PLP1 *Plp*^ΔTEC^ mice exhibited clear indications of peripheral antigen encounter. Thus, despite an identical thymocyte composition as in TCR-PLP1 *Plp*^KO^ mice (see Figures 5B,C), there was a marked reduction in the overall size of the peripheral CD4 T cell population in TCR-PLP1 *Plp*^ΔTEC^ mice (Figure 7A). Although this resembled a similar CD4 T cell reduction in TCR-PLP1 *Plp*^WT^ mice, *Plp*^ΔTEC^ mice had a substantially higher percentage of TCR-PLP1^+^ cells among peripheral CD4 T cells (47.9 ± 5.1 versus 8.9 ± 0.8%; *P* < 0.001). The proportion of Foxp3^+^ T_reg_ cells among TCR-PLP1^+^ CD4 T cells in *Plp*^ΔTEC^ mice was as low as in fully PLP-deficient mice, suggesting that PLP expression in TECs was crucial for the establishment of the prominent peripheral TCR-PLP1^+^Foxp3^+^ T_reg_ population in TCR-PLP1 *Plp*^WT^ mice. By contrast, the CD25^–^ subset of TCR-PLP1^+^ cells from *Plp*^ΔTEC^ mice functionally resembled the corresponding population in *Plp*^WT^ mice with respect to being refractory to antigenic stimulation *in vivo* (Figure 7B), indicating that anergy induction occurred independent of thymic PLP encounter. Consistent with this, the anergy marker FR4 was similarly elevated on Foxp3^–^CD25^–^TCR-PLP1^+^ cells from both *Plp*^ΔTEC^ and *Plp*^WT^ mice (Figure S2 in Supplementary Material). These data showed that in the absence of PLP expression in TECs, peripheral tolerance toward PLP epitope #1 appeared to operate *via* deletional mechanisms and anergy induction rather than T_reg_ conversion.

### Peripheral Tolerance Induction through Presentation of “Non-Hematopoietic” PLP by DCs

Ablation of thymic PLP expression in *Plp*^ΔTEC^ mice revealed that “downstream” of the thymus, peripheral antigen encounter could shape the fate of TCR-PLP1 CD4 T cells. To ask whether presentation of I-A^b^/PLP_11–19_ ligands in the periphery was DC dependent, we transferred CFSE-labeled TCR-PLP1 cells into DC-sufficient or DC-deficient *Plp*^WT^ mice. Whereas transferred cells vigorously proliferated in control recipients, they failed to do so in ΔDC mice, indicating that antigen presentation by DCs was crucially involved (Figure 7C).

To address whether the display of PLP epitope #1 by peripheral DCs reflected the presentation of endogenously derived PLP or required the uptake and presentation of PLP from a non-hematopoietic source, we performed analogous experiments with bone marrow (BM) chimeric recipients (Figure 7D). Whereas TCR-PLP1 cells underwent proliferation when transferred into MHCII^+/+^ → *Plp*^WT^ control chimeras, they remained quiescent in MHCII^–/–^ → *Plp*^WT^ chimeras, consistent with a requirement for antigen presentation by DCs. Importantly, transferred cells likewise failed to proliferate in MHCII^+/+^ → *Plp*^KO^ chimeras, establishing that PLP from a non-hematopoietic source was crucially required.

Considering that in *Plp*^WT^ mice, PLP was concomitantly presented intrathymically by mTECs as well as in the periphery by DCs, we wondered in how far the peripheral composition and functionality of TCR-PLP1^+^ cells was shaped by thymic output versus peripheral tolerance induction. The striking phenotypic similarity between DC-sufficient and DC-deficient TCR-PLP1 *Plp*^WT^ mice (compare Figures 6A,B) also applied to the periphery (Figure 7A). Specifically, total CD4 cells were reduced to the same extent, and the comparably small populations of TCR-PLP1^+^ cells in both genotypes similarly segregated into Foxp3^+^ T_regs_ and Foxp3^–^CD25^–^ cells, suggesting that the overall composition of the TCR-PLP1^+^ CD4 T cell pool was mostly shaped by thymic output. Interestingly, though, CD25^–^ cells from ΔDC mice proliferated upon adoptive transfer into *Plp*^WT^ mice (Figure 7B), indicating that the anergic state of these cells in DC-sufficient mice is induced and/or maintained by peripheral antigen encounter on DCs.

Our findings suggested that the lack of thymic negative selection in TCR-PLP1 *Plp*^ΔTEC^ mice could be largely compensated by DC-mediated peripheral deletion and anergy induction, while the “normal” formation of a PLP-specific peripheral T_reg_ compartment appeared to depend on thymic T_reg_ induction by mTECs. Given this differential degree of redundancy between thymic and peripheral antigen encounter with respect to recessive versus dominant tolerance mechanisms (deletion and anergy versus T_reg_ induction, respectively), we sought evidence for a defect in dominant tolerance to PLP in the polyclonal repertoire of non-TCR transgenic *Plp*^ΔTEC^ mice. To do so, we adoptively transferred naïve TCR-PLP1 cells into *Plp*^ΔTEC^ or *Plp*^WT^ mice simultaneously to “EAE-immunization” with PLP_11–20_. This resulted in the development of EAE in both genotypes. However, the disease course was more severe in mice lacking thymic PLP expression, consistent with a “less robust” state of tolerance, possibly owing to lack or diminution of PLP-specific T_reg_ cells (Figure 7E).

## Discussion

The present study establishes a number of salient features of tolerance to PLP in BL/6 mice. The fine mapping of the protein domains against which the uncensored CD4 T cell repertoire of PLP-deficient mice reacts identified three core I-A^b^-restricted epitopes. PLP-sufficient mice failed to mount a response against epitopes #1 and #3, yet reacted toward epitope #2. This correlated tightly with the encephalitogenic potential of the respective peptides when administered according to the standard EAE protocol, indicating that the extent of CD4 T cell tolerance varied between epitopes. Interestingly, epitope #2 overlaps with a peptide that is commonly used to elicit EAE in SJL/J (H-2^s^) mice, but was shown to bear encephalitogenic potential also in BL/6 mice (28, 29).

The precise determinants underlying the differential degree of tolerance of BL/6 mice to the I-A^b^ restricted epitopes #1 (PLP_11–19_) and #3 (PLP_240–248_) on the one hand and epitope #2 (PLP_174–182_) on the other hand remain to be established. In SJL mice, a similar lack of tolerance to the I-A^s^-restricted encephalitogenic peptide PLP_139–151_, at least as far as central tolerance induction is concerned, is readily explained by the fact that expression of PLP in TECs is confined to the DM20 splice variant that lacks the amino acid residues 116–151 (8, 30). However, such a splicing-related “hole in the proteome” cannot account for the leakiness of central tolerance to epitope #2, since all three I-A^b^-restricted PLP epitopes are contained within the intrathymically expressed DM20 protein isoform and should therefore be available for processing and presentation in stoichiometric amounts. Hence, the most likely explanation for the lack of central tolerance to epitope #2 might be that this peptide is a weaker I-A^b^ binder than epitopes #1 and #3. A scenario whereby escape from central tolerance due to weak or unfavorable MHCII binding explains the autoimmune association of a given self-epitope is reminiscent of what has been suggested regarding the major insulin-epitope recognized by diabetogenic CD4 T cells in NOD mice (H-2^q7^) (31, 32) and the encephalitogenic MBP epitope MBP_1–11_ in H-2^u^ mice (33). An experimental verification of the predicted I-A^b^-binding hierarchy of PLP epitopes will be necessary to further substantiate this notion.

Weak I-A^b^ binding would also offer an explanation why epitope #2 appears to be insufficiently presented in the periphery and therefore “ignored” by specific CD4 T cells, whereas epitope #1 is constitutively available for T cell recognition and, under *steady state* conditions, peripheral tolerance induction. Despite the caveat that our study is limited to the fate of individual transgenic TCRs, we deem it likely that escape from thymic censorship and “ignorance” in the periphery is representative of the fate of a sizeable fraction of epitope #2-specific CD4 T cells in the polyclonal repertoire. Thus, despite intrathymic expression of PLP, EAE can be elicited in BL/6 mice by immunogenic delivery of this determinant, indicating that a sufficiently large pool of “naïve” epitope #2 reactive CD4 T cells exists.

Breeding onto a Rag-deficient background, although only modestly increasing the frequency of clonotype-positive cells, rendered TCR-PLP2 mice susceptible to spontaneous EAE development. This is reminiscent of a similar phenomenon in H-2^u^ mice carrying an MBP_1–11_-specific TCR (34). In the latter model, it was shown that T_reg_ cells, whose differentiation requires endogenous TCRα rearrangements conferring specificity to unknown self-antigens in addition to the MBP-specificity, are crucial to prevent disease (35–37). Where and how TCR-PLP2 cells are primed at the onset of EAE in TCR-PLP2 *Rag1*^–/–^ mice remain to be established. Yet, the spontaneous occurrence of disease in these animals illustrates that the presumed “invisibility” of PLP epitope #2 and the ensuing “ignorance” of specific CD4 T cells reflects a rather fragile tolerance state that may depend upon a delicate balance of active control mechanisms.

In contrast to the observations with epitope #2, immunization with epitopes #1 or #3 failed to promote a recall response and lacked encephalitogenic potential, and epitope #1 reactive TCR transgenic CD4 T cells were subject to central tolerance induction in the thymus. We consider it likely that central tolerance induction generally applies to epitope #1-specific CD4 T cells exceeding a certain affinity threshold and by inference also to epitope #3 reactive T cells. To which extent this occurs through clonal deletion or clonal diversion into the T_reg_ lineage for a given epitope remains to be established. In this regard, the precise mapping of I-A^b^ restricted epitopes paves the way for future investigations at the polyclonal repertoire level with MHCII-tetramer reagents.

Our findings establish several molecular and cellular details of how PLP is expressed and presented for central tolerance. First, the conditional ablation of PLP in TECs unequivocally established that intrathymic expression by epithelial cells was essential, excluding a scenario whereby extrathymically derived PLP may reach the thymus as blood-borne antigen or through import by migratory DCs (38–40). Second, Aire was crucially required for central tolerance induction in TCR-PLP1 cells. This was somewhat unexpected, given that intrathymic PLP transcription, in contrast to “canonical” promiscuous gene expression, is not confined to Aire^+^ MHCII^hi^ mTECs, but occurs at comparable levels also in their Aire^neg/lo^ MHCII^lo^ immature precursors as well as in cTECs (8, 41). In *Aire*^–/–^ mTECs, PLP transcripts are reduced by about fivefold (41), possibly falling below a critical threshold for central tolerance induction. However, tolerogenic functions of Aire beyond the promotion of promiscuous gene expression, for instance through regulation of chemokine expression, cellular differentiation, or antigen presentation may also contribute (42–46). These considerations notwithstanding, the “late” deletion of TCR-PLP1 cells at the CD4 SP stage is consistent with PLP expression in cTECs being irrelevant. Third, endogenously expressed PLP is directly presented by TECs, indicating that there is no requirement for “antigen handover” from expressing TECs to thymic DCs (24). The latter has not only been reported for certain “neo-antigens” expressed in mTECs (47) but was also suggested to occur for physiologically expressed self-antigens (48, 49). However, ablation of DCs did not interfere with central tolerance induction in the TCR-PLP1 model, whereas conversely, reduced direct presentation by mTECs in the C2TAkd system had a significant effect. Of note, the direct presentation of TEC-derived PLP contrasts with what has been reported for an epitope of another Myelin autoantigen, MBP, whose tolerogenic display in the thymus required hematopoietic APC-mediated presentation of MBP of non-hematopoietic origin (50). Direct MHCII-restricted presentation of endogenous antigens by TECs can be facilitated through routing of intracellular material into autophagosomes (25, 51, 52), but whether this pathway is involved in central tolerance to PLP remains to be addressed.

Bypassing or eliminating the thymic checkpoint through adoptive transfer of TCR-PLP1 cells or conditional deletion of PLP in TECs, respectively, revealed that PLP epitope #1 is constitutively “visible” to specific CD4 T cells in the periphery. Intriguingly, whereas DCs were dispensable for central tolerance, the presentation of PLP in the periphery critically involved DCs, as evident from the failure of adoptively transferred TCR-PLP1 cells to proliferate in ΔDC recipients or MHCII^–/–^ → *Plp*^WT^ BM chimeras. The exact source of PLP that is presented by DCs in the periphery remains to be identified, but the lack of proliferation of adoptively transferred TCR-PLP1 cells in MHCII^+/+^ → *Plp*^KO^ BM chimeras indicates a non-hematopoietic origin. Along these lines, it will be interesting to establish the anatomical location and exact identity of the DC subset(s) by which PLP is peripherally acquired and presented; these cells obviously lack the capacity to efficiently migrate to the thymus and induce central tolerance.

A general defect in intrathymic expression of tissue-restricted antigens, for instance in *Aire*- or *Fezf2*-deficient mice (53, 54), or the selective interference with intrathymic expression of individual genes, as is the case for the eye autoantigen IRBP (55), can precipitate spontaneous autoimmunity. By contrast, the deficiency in central tolerance to epitope #1 in *Plp*^ΔTEC^ mice neither resulted in spontaneous EAE nor increased the susceptibility to “induced” EAE, irrespective of whether or not these mice were TCR transgenic. This suggests a substantial degree of “functional” redundancy between mTEC-mediated central tolerance and DC-mediated peripheral tolerance to PLP. At the mechanistic level, however, our data point toward a differential contribution of central and peripheral tolerance induction to recessive versus dominant modes of tolerance, that is, clonal deletion and anergy versus Treg induction, respectively. On the one hand, there was a similar peripheral CD4 T cell reduction in TCR-PLP1 mice on both *Plp*^WT^ as well as *Plp*^ΔTEC^ background, indicating that deletional tolerance mechanisms also operate in the periphery. Likewise, the anergic state of the Foxp3^–^ subset of TCR-PLP1^+^ cells in the periphery was independent of thymic PLP expression. On the other hand, however, the “normal” formation of a PLP-specific peripheral T_reg_ compartment appeared to depend upon thymic T_reg_ induction by mTECs. Consistent with impaired dominant tolerance to PLP in the absence of central tolerance induction, *Plp*^ΔTEC^ mice exhibited a more severe EAE course than *Plp*^WT^ mice when receiving adoptively transferred naïve TCR-PLP1 at the time of “active EAE immunization” with PLP_9–20_.

TCR-PLP1 ΔDC mice represent an informative model regarding the question to which extent the PLP-specific CD4 T cell repertoire is shaped by thymic output as opposed to peripheral encounter of self-antigen: regardless of the absence of DCs, the peripheral CD4 T cell pool in these mice essentially phenocopied that of DC-sufficient mice, arguing for a prevailing influence of intrathymic tolerance induction. However, the maintenance and/or induction of the anergic state of the Foxp3^–^ subset of TCR-PLP1^+^ cells in the periphery required antigen (re-)encounter on peripheral DCs.

In sum, our work provides a comprehensive analysis of the cellular and molecular determinants that specify self-tolerance to PLP, the major myelin protein, in BL/6 mice. Our findings reveal distinct levels of redundancy or non-redundancy in terms of central versus peripheral tolerance induction and how these relate to EAE susceptibility. The definition of the I-A^b^-restricted CD4 T cell epitopes of PLP in conjunction with the comprehensive analysis of the tolerance modes that apply to these epitopes will lead the way to a deeper understanding of how repertoires directed against different epitopes of the very same CNS autoantigen may cooperate during disease induction and resolution.

## Materials and Methods

### Mice

MHC class II^–/–^ (*H2-Ab1*^–/–^) (56), C2TAkd (27), ΔDC (26), *Plp*^KO^ (20), and *Aire*^–/–^ (57) mice have been described previously. The gene encoding for PLP is located on the X chromosome. We did not observe any differences in the composition and phenotype of lymphoid compartments between TCR-PLP1 or TCR-PLP2 transgenic PLP-sufficient females (*Plp*^+/+^) and males (*Plp*^+/y^) or between PLP-deficient females (*Plp*^–/–^) or males (*Plp*^–/y^). Therefore, female and male mice were included into the PLP-sufficient or -deficient groups and their genotype indicated as *Plp*^WT^ or *Plp*^KO^. *Plp*^ΔTEC^ mice were obtained by crossing Foxn1-Cre (58) mice to mice carrying a conditional *Plp* allele in which exon 3 is flanked by loxP sites (*Plp*^fl^) (59). C57BL/6 mice were purchased from Charles River. Mice were maintained under specific pathogen-free conditions in individually ventilated cages. All phenotypic analyses were performed in young adult mice at 4–5 weeks of age. Animal studies and procedures were approved by local authorities (Az 7-08 and 142-13).

To generate TCR transgenic mice, we used rearranged V(D)J regions of TCRs from PLP epitope #1 or #2-specific T cell hybridomas (D9-119-2 or A43-11-5, respectively). Hybridoma D9-119-2 (TCR-PLP1) was obtained by immunizing *Plp*^KO^ mice with peptide PLP_1–25_, repeated re-stimulation of draining LN cells with PLP_9–20_ and subsequent fusion with BW5147 cells. Its TCRα chain contains a rearrangement of Trav9d-3.201 (Vα3.2) and Traj34-201, the TCRβ chains harbors a Trbv19-201 (Vβ6), Trbd1, and Trbj1-1 rearrangement. Hybridoma A43-11-5 (TCR-PLP2) was obtained by immunizing *Plp*^KO^ mice with peptide PLP_160–184_, repeated re-stimulation of draining LN cells with PLP_172–183_, and subsequent fusion with BW5147 cells. Its TCRα chain contains a Trav14-1-201 (Vα2) and Traj23-201 rearrangement, the TCRβ chain harbors a Trbv31-01 (Vβ14), Trbd1, and Trbj1-1 rearrangement. TCRα and TCRβ rearrangements from the D9-119-2 and A43-11-5 hybridomas were PCR amplified and cloned into the cassette vectors pTαcass and pTβcass as described before (60). Transgenic mice were generated by injection of linearized DNA into pronuclei of C57BL/6 zygotes.

### Antibodies and Flow Cytometry

FITC-conjugated monoclonal antibodies (mAbs) to Vα3.2 (RR3-16); PE-conjugated mAbs to Vβ6 (RR4-7), Vα2 (B20.1), and Vβ14 (14-2); CyChrome-conjugated mAbs to CD8 (53-6.7), PE-Cy7-conjugated streptavidin and mAbs to CD25 (PC61) and FR4 (12A5); APC-Cy7-conjugated mAbs to CD4 (GK1.5); biotin-conjugated mAbs to CD8 (53-6.7), CD4 (GK 1.5), B220 (RA3-6B2) and Vα2 (B20.1); APC-conjugated mAbs to Foxp3 (FJK-16s), CD45.2 (104); Pacific Blue-conjugated mAbs to CD45.1 (A20) were from BioLegend or eBiosciences, respectively; flow cytometric analyses were performed on a FACSCanto II (BD) using FACSDiva (BD) and FlowJo (Tree Star) software.

### Peptides and Antigens

Purified human PLP (100% identical to mouse PLP) was prepared as described (7). 25-mer peptides were synthesized in the central core facility of the DKFZ (8). 12-mer peptides were synthesized in the central core facility of the DKFZ or purchased from BioTrend.

### Epitope Prediction and *In Silico* Analysis

The IEDB tool (http://www.iedb.org/) combines various machine learning algorithms for peptide epitope prediction in the context of a given MHC haplotype. The full-length PLP amino acid sequence was analyzed using the IEDB tool in the default “consensus” setting for prediction methods (21, 22). A lower score indicates better binding to MHCII. The theoretical IC_50_ values were obtained with the SMM-align predictor (23).

### Immunization and T Cell Proliferation Assays

Mice were immunized with 100 µg purified PLP or 50 µg peptide emulsified in complete Freund’s adjuvant (volume/volume, 1/1). After 9 days, popliteal and inguinal lymph node cells were harvested and cultured for 72 h in triplicate at a concentration of 4 × 10^5^ cells/well in round-bottomed, 96-well plates in serum-free medium (HL-1; BioWhittaker) in the presence or absence of titrated amounts of peptide or 5 µg/ml of the respective peptide for epitope mapping. Proliferation was measured by incorporation of 3H-thymidine, added for the last 24 h of culture at a concentration of 1 μCi/well. Only mice with comparable control responses to the complete Freund’s adjuvant component PPD were included in the analyses. Proliferation indices were calculated as ^3^H-Thymidin-incorporation in the presence of specific peptide divided by background proliferation w/o peptide. For direct *ex vivo* proliferation assays, 4 × 10^5^ T cells and 3 × 10^4^ irradiated (3,000 rad) BM-derived DCs were cultured in the presence of increasing peptide concentrations.

### BM Chimeras

Bone marrow was depleted of B and T cells using biotinylated mAbs to B220, CD8, and CD4 and streptavidin MACS beads (Miltenyi Biotech). Female recipient mice were irradiated with 2 × 550 rad and reconstituted with 5 × 10^6^ BM cells.

### Adoptive T Cell Transfer

TCR-PLP1 or TCR-PLP2 CD4^+^ T cells were isolated from pooled lymph nodes and spleen by cell sorting or by depletion of CD25^+^ cells with biotin-conjugated anti-CD25 and anti-SAV MicroBeads followed by positive enrichment with anti-CD4 microbeads (Miltenyi Biotech). T cells were labeled with CFSE (Life Technologies) and 5 × 10^6^ T cells were injected i.v. into the lateral tail vein of congenic recipient mice. After 4 days, spleen or lymph node cells of recipient mice were analyzed by flow cytometry.

### Th1 and Th17 Polarization

Naive CD4^+^ T cells were prepared from TCR-PLP1 *Plp*^KO^ mice using the mouse CD4 T cell isolation kit (Miltenyi Biotech). For Th1 polarization, cells were incubated for 5 days in anti CD3 (2C11) (5 µg/ml) coated 12-well plates (1 × 10^6^ cells/well) in the presence of recombinant IL-2 (5 ng/ml), recombinant IL-12 (10 ng/ml), anti-CD28 mAb (37.51) (3 µg/ml), and anti-IL-4 mAb (11B11) (10 µg/ml). For Th17 polarization, cells were stimulated in the presence of IL-6 (50 ng/ml), IL-1β (10 ng/ml), TGF-β1 (1 ng/ml), anti-CD28 (5 µg/ml), anti-IL-4 (10 µg/ml), anti-IFN-γ (XMG1.2) (10 µg/ml), and anti-IL-2 (JES6-1A2) (10 µg/ml). To verify successful polarization, cells were re-stimulated on day 5 with PMA and ionomycin for 5 h and stained intracellularly for expression of lineage-specific effector cytokines (IFN-γ and IL-17).

### *In Vito* Suppression Assay

TCR-PLP1^+^CD25^+^ cells were sorted from TCR-PLP1 *Plp*^WT^ mice (CD45.2) and co-cultured at different ratios with 2 × 10^4^ CFSE-labeled TCR-PLP1^+^CD25^–^ responder cells (CD45.1) from TCR-PLP1 *Plp*^KO^ mice in the presence of 2 × 10^5^ irradiated spleen APCs and 5 µg/ml peptide PLP_9–20_. After 4 days, CFSE dilution in gated CD45.1^+^ cells was analyzed by flow cytometry.

### Experimental Autoimmune Encephalomyelitis

For “active EAE” induction, mice were immunized subcutaneously at three sites on the flanks with 200 µg peptide PLP_9–20_, PLP_172–183_, or PLP_238–249_ in a total of 200 µl PBS/CFA emulsion (1:1). Pertussis toxin (400 ng) was given on days 0 and 2. In some experiments, mice received 5 × 10^6^ naïve TCR-PLP1 cells 6 h prior to the immunization. Signs of EAE were scored as follows: 0, none; 0.5, partial loss of tail tonus; 1, limp tail; 1.5, wobbly gait and limb tail; 2.5, paralysis of one hind leg; 3, hind-limb paralysis on both sides; 3.5, hind-limb and partial front limbparalysis; 4, complete hind-limb and forelimb paralysis; 5, moribund. Disease incidence and scores were measured daily. For “passive EAE” induction with Th1 or Th17 cells, *Rag*1^–/–^ *Plp*^WT^ recipients were injected i.v. with 5 × 10^6^ *in vitro* polarized TCR-PLP1 cells. Scoring was carried out as described above.

### Preparation of Thymic Stromal Cells and Quantitative PCR

Thymic stromal cells were isolated as described (61). TEC subtypes were classified according to the following surface phenotypes: cTECs, CD45^−^EpCAM^+^Ly51^+^CD80^−^; mTEC^hi^, CD45^−^EpCAM^+^Ly51^−^CD80^hi^MHCII^hi^; mTEC^lo^, and CD45^−^EpCAM^+^Ly51^−^CD80^lo^MHCII^lo^. RNA was isolated from sorted cells using the miRNAeasy kit including a DNase digest (Qiagen) and reverse transcribed using the iScript cDNA synthesis kit (Bio-Rad). PCR reactions were performed in duplicates on a CFX96 real-time thermal cycler (Bio-Rad) using the Ssofast EvaGreen Supermix (Bio-Rad). Fluorescence was recorded at the annealing step and relative expression levels were calculated with the comparative Ct method, using HPRT as housekeeping gene for normalization. Primers: *Plp*-forward, GGGCTTGTTAGAGTGTTGTGC; *Plp*-reverse, GAAGAAGAAAGAGGCAGTTCCA; *Hprt*-forward, TGAAGAGCTACTGTAATGATCAGTCAAC; *Hprt*-reverse, AGCAAGCTTGCAACCTTACCA.

### Statistical Analysis

Unless indicated otherwise, statistical significance was assessed using the two-tailed unpaired Student’s *t*-test with Welch’s correction for unequal variances.

## Ethics Statement

Animal studies and procedures were approved by local authorities (Az 7-08, 142-13, 88-17).

## Author Contributions

MH and LK designed experimental strategies. LK and BK mapped PLP epitopes. JW and MH generated and analyzed TCR-PLP1 mice. LW, MH, and CF generated and analyzed TCR-PLP2 mice. GJ performed mRNA expression analyses. K-AN and HW provided *Plp*^KO^ and *Plp*^floxed^ mice. LK and MH wrote the manuscript.

## Conflict of Interest Statement

The authors declare that the research was conducted in the absence of any commercial or financial relationships that could be construed as a potential conflict of interest.
