# Supplementary figures and images for: Epitope-Specific Tolerance Modes Differentially Specify Susceptibility to Proteolipid Protein-Induced Experimental Autoimmune Encephalomyelitis
Source: Front Immunol. 2017 Nov 9;8:1511. doi: 10.3389/fimmu.2017.01511 (PMC5684123; doi:10.3389/fimmu.2017.01511)

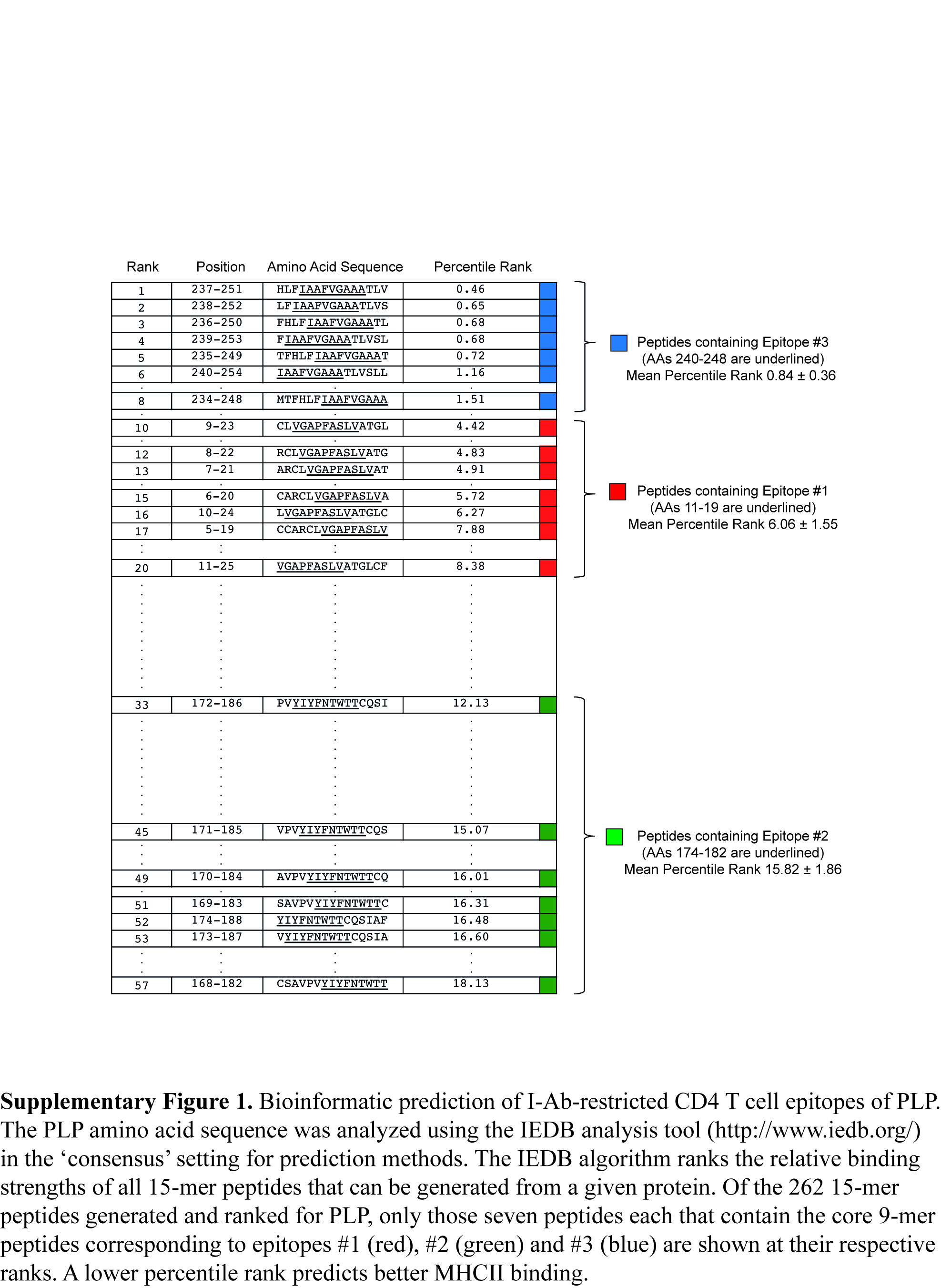

Supplement: Supplementary file 1 [file image_1.tif]

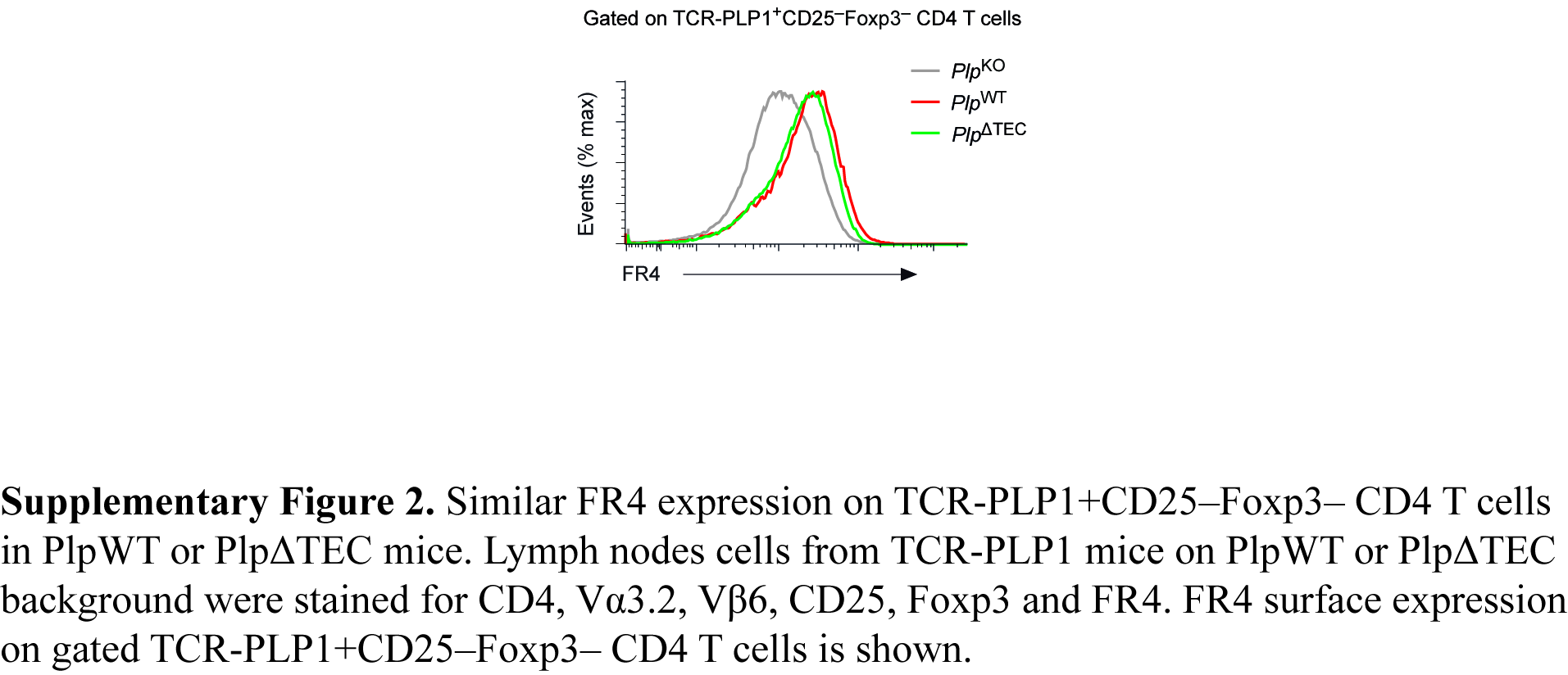

Supplement: Supplementary file 2 [file image_2.tif]
